# Mucosal Healing Did Not Predict Sustained Clinical Remission in Patients with IBD after Discontinuation of One-Year Infliximab Therapy

**DOI:** 10.1371/journal.pone.0110797

**Published:** 2014-10-20

**Authors:** Cong Dai, Wei-Xin Liu, Min Jiang, Ming-Jun Sun

**Affiliations:** Department of Gastroenterology, First Affiliated Hospital, China Medical University, Shenyang City, Liaoning Province, China; INSERM, France

## Abstract

**Aim:**

To assess the endoscopic activity and Clinical activity after a one-year period of infliximab therapy and to evaluate the association between mucosal healing and need for retreatment after stopping infliximab in patients with Inflammatory bowel disease (IBD).

**Methods:**

The data from 109 patients with Crohn’s disease (CD) and 107 patients with Ulcerative colitis (UC) received one-year infliximab were assessed. The primary endpoint of the study was the proportion of clinical remission, mucosal healing and full remission in IBD after the one-year period of maintenance infliximab therapy. The secondary endpoint was the frequency of relapses in the next year.

**Results:**

A total of 84.4% (92/109) CD patients and 81.3% (87/107) UC patients achieved clinical remission, 71.56% (78/109) of CD patients and 69.16% (74/107) of UC patients achieved mucosal healing, 56.88% (62/109) of CD patients and 54.21% (58/107) of UC patients achieved full remission at the end of the year of infliximab therapy. Infliximab therapy was restarted in the 10.19% (22/216) patients (13 CD, 9 UC) who achieved mucosal healing, and 13.89% (30/216) patients (18 CD, 12 UC) who achieved clinical remission and 6.48% (14/216) patients (8 CD, 6 UC) who achieved full remission had to be retreated within the next year. Neither clinical remission nor mucosal healing was associated with the time to restarting Infliximab therapy in IBD.

**Conclusion:**

Mucosal healing did not predict sustained clinical remission in patients with IBD in whom the infliximab therapies had been stopped. And stopping or continuing infliximab therapy may be determined by assessing the IBD patient’s general condition and the clinical activity.

## Introduction

Inflammatory bowel disease (IBD) is a chronic recurrent disease, which mainly consists of ulcerative colitis (UC) and Crohn’s disease (CD) [1]. And it is a growing worldwide health burden especially in many developing countries. Although not completely defined, the aetiopathology of IBD is thought to be a consequence of immune dysregulation, impaired mucosal integrity, enteric bacterial dysbiosis and genetic susceptibility factors [2], [3], [4]. The aims of treatment in IBD are to induce and maintain remission, to improve quality of life and to prevent the development of complications and the need for surgery. Now the pharmaceutical therapies have included corticosteroids, 5-aminosalicylic acids (5-ASAs), immunomodulators such as azathioprine, 6-mercaptopurine (6-MP), methotrexate, and biological therapy such as infliximab, adalimumab [5], [6].

Colonoscopies play an important role in the diagnosis, management and monitoring of IBD. Now the most important goals in the treatment of IBD is mucosal healing, because some studies have demonstrated that mucosal healing can alter the course of IBD due to its association with sustained clinical remission and reduced rates of hospitalization and surgery [7]. And mucosal healing at the time of treatment withdrawal may predict better outcomes in IBD [8].

The endoscopy provides a direct evaluation of the mucosal lesions in IBD and intestinal activity may be quantified by indices of endoscopic activity, but a clear definition of mucosal healing is still lacking. Most of the clinical trials define mucosal healing as the total disappearance of mucosal ulcerations. The Simplified Endoscopic Activity Score for Crohn’s Disease (SES-CD) is a relatively easy tool to evaluate the endoscopic activity in CD [9], and the Mayo endoscopic score is common indices to evaluate the endoscopic activity in UC [10], [11]. The weaknesses of these endoscopic activity indices include the absence of a clear definition and the lack of validation of mucosal healing [12]. At the same time, routine endoscopic follow-up is recommended for all IBD patients who have achieved clinical remission with medical therapy; for those with persistent complaints, in order to rule out post-inflammatory irritable bowel syndrome; for those still within their first year after surgery; and for those who are stopping biological therapies but continuing immunosuppressants [13], [14].

The introduction of monoclonal anti-TNF-α drugs revolutionised the management of IBD. Although the precise mechanism of action is unknown, it is thought that anti-TNF-α drugs cause apoptosis of inflammatory cells carrying membrane-bound TNF-α, an important cytokine in the pathogenesis of IBD. And anti-TNF-α drugs have proven their efficacy in inducing and maintaining clinical and endoscopic remission in both CD and UC. For example, Good data exist demonstrating the efficacy of anti-TNF-α drugs for inducing and sustaining remission for patients with moderate to severely CD, with approximately 60% of patients showing overall clinical improvement [15], [16]. Long-term data have shown infliximab to be beneficial, in initial responders, over a median follow-up period of 4.6 years [17]. The efficacy of anti-TNF-α drugs in the treatment of UC is less impressive than their effect in CD. The ACT UC Trials were large, multicentre RCTs that compared infliximab with placebo in the treatment of moderate to severe active UC [13]. There was a modest, but significant benefit in clinical improvement over placebo.

According to the Consensus Statement on Diagnoses and Treatment of IBD in China, biological therapies (infliximab approved for the treatment of CD and UC) have to be discontinued after a one-year maintaining clinical remission treatment period. The endoscopic healing of the mucosa is commonly evaluated at the end of the one-year treatment period with biological therapies. In the present study, we want to assess the endoscopic activity and the rate of mucosal healing after a one-year period of infliximab therapy and to evaluate how the endoscopic findings of the mucosa predict the frequency of relapses and the need for restarting infliximab therapy.

## Materials and Methods

### Study design

This was a prospective observational study conducted in the Department of Gastroenterology, First Affiliated Hospital, China Medical University between January 2010 and December 2013. The study was approved by China Medical University Regional and Institutional Committee of Science and Research Ethics and by the Regional and Institutional Human Medical Biological Research Ethics Committee of China Medical University. All participants have provided their written informed consent to participate in our study. The ethics committees have approved this consent procedure. We have also obtained informed consent from the next of kin, caretakers, or guardians on behalf of the minors/children enrolled in our study.

The analysis focused on patients who underwent an ileocolonoscopy before and after the one-year maintenance infliximab therapy and in whom infliximab therapy were discontinued at the end of the year. Endoscopies were performed by two experienced gastroenterologists after stopping the one-year infliximab therapy.

One hundred and nine CD patients (59 females, 41 males, mean disease duration at the beginning of biological therapy: 6.2 years) and 107 UC patients (59 females, 48 males, mean disease duration at the beginning of biological therapy: 11.4 years) were prospectively followed up in this study. Twelve CD patients (8 females, 4 males, mean disease duration at the beginning of biological therapy: 5.8 years) and 8 UC patients (5 females, 3 males, mean disease duration at the beginning of biological therapy: 9.9 years) were lost in the follow-up. There was no significant difference about the basic clinical characteristics of patients between included and not included in the study. All patients received maintenance infliximab therapy for one year in accordance with the Consensus Statement on Diagnoses and Treatment of IBD in China. CD disease phenotypes were determined according to the Montreal Classification [18]. These patients received the last dose of infliximab therapy at least 3 month before the repeated one-year therapy. Seventy-three patients were naive to biological therapy (did not receive biological therapy before the one-year treatment period analyzed in the study) in the CD group, and 68 patients in the UC group. The concomitant immunosuppression during the induction therapy was corticosteroids in 100 (CD: 66, UC: 34) and azathioprine in 66 (CD: 45, UC: 21) patients. The clinical characteristics of the patients are presented in Table 1. Patients’ data regarding smoking status, previous appendectomy, perianal involvement, presence of extraintestinal manifestation, outcome of induction therapy, previous surgical procedures, and previous biological therapy were collected.

**Table 1:** Demographic and clinical characteristics of patients.

	CD patients (*n* = 109)	UC patients (*n* = 107)
Female/male	68/41	59/48
Mean age at diagnosis (yr)	26 (14–63)	31 (12–57)
Mean age at the beginning of infliximab therapy (yr)	32 (19–64)	42 (15–65)
Age at diagnosis		
<20 years (A1)	15	17
20–40 years (A2)	73	68
>40 years (A3)	21	22
Location		
Ileal (L1)	20	-
Colonic (L2)	31	-
Ileocolonic (L3)	56	-
Upper GI (L4)	2	-
Proctitis	-	0
Left-sided colitis	-	64
Extensive colitis	-	43
Behaviour		
Inflammatory (B1)	53	-
Stricturing (B2)	17	-
Penetrating (B3)	35	-
Perianal manifestation	41	-
Extraintestinal manifestation	39	12
Surgery before infliximab therapy	23	5
Previous biological therapy	17	3
Median CDAI/pMayo at the start of infliximab therapy	328	9.8
Median CRP level at the start of infliximab therapy (mg/l)	13.8	11.2
Current smokers	74	21
Appendectomy	13	1

### Assessment of clinical and endoscopic remission

Clinical activities, as determined by the Crohn’s Disease Activity Index (CDAI) [19] in CD and by the Mayo score [10] in UC, were calculated at the end of infliximab therapy when the endoscopic assessment was performed, while partial Mayo scores were calculated when infliximab therapy needed to be restarted. Clinical remission was defined as a CDAI of <150 points and a Mayo score of <2 points. Sustained clinical remission was defined as a stable, steroid-free clinical remission during the 1-year follow-up period. The definition of relapse and indication for restarting biologicals were an increase of >100 points in CDAI and a CDAI of >150 points and a partial Mayo score of >3 points.

The endoscopic severity of CD was quantified with SES-CD in CD [8] and with Mayo endoscopic subscore in UC [10]. The endoscopic scores were prospectively assessed by two investigators (Min Jiang and Ming-Jun Sun). Mucosal healing was defined using the endoscopic indices as SES-CD between 0 and 3 and Mayo endoscopic subscore as 0.

### Endpoints

Data collection and analysis were performed at the Department of Gastroenterology, First Affiliated Hospital, China Medical University. The primary endpoint of the study was the proportion of mucosal healing in IBD after the one-year period of maintenance infliximab therapy. The secondary endpoint was the frequency of relapses in the next year.

### Statistical analysis

Variables were tested for normality using Shapiro-Wilk’s W test. The χ^2^-test and logistic regression analysis were used to assess the association between categorical clinical variables and clinical/endoscopic outcomes. The variables analyzed were gender, disease duration, active smoking, appendectomy, location/extent, behavior, associated perineal disease, extraintestinal manifestations, previous surgery, previous biological therapy, clinical activities, CRP levels, and outcomes of induction therapy. The difference between patients with mucosal healing and those who failed to achieve endoscopic remission was assessed by chi-square or Fisher’s exact tests. Kaplan-Meier survival curves were plotted for analysis with the Log-Rank and Breslow tests. For the statistical analysis, SPSS 11.5 was used. And P<0.05 was considered significant.

## Results

### Clinical activity of IBD after the one-year period of infliximab therapy

The median CDAI was 72 (interquartile range: 36.8–97.5) (P*<*0.01) and the partial Mayo score was 1.4 (interquartile range: 0–5.2) (*P<*0.01) at the end of the treatment period. A total of 92/109 patients with CD (84.4%) and 87/107 with UC (81.3%) achieved clinical remission at the end of the year of infliximab therapy.

### Endoscopic activity of IBD before and after the one year period of infliximab therapy

Colonoscopies reached the terminal ileum in each patient. The median values of the SES-CD and the Mayo endoscopic subscores significantly improved after infliximab therapy [18 (interquartile range: 11–25) vs 6 (interquartile range: 2–11), *P<*0.01, and 3 (interquartile range: 2–4) vs 1 (interquartile range: 0–3), *P<*0.01]. Mucosal healing was achieved in 71.56% (78/109) of CD patients and 69.16% (74/107) of UC patients. Full remission – both mucosal healing and clinical remission - was achieved in 56.88% (62/109) of CD patients and 54.21% (58/107) of UC patients.

### The frequency of relapses in the next year

During the next year follow up period, 13.89% (30/216) of patients (18 CD, 12 UC) in the clinical remission group and 6.48% (14/216) patients (8 CD, 6 UC) in the full remission group had to be retreated. In CD, infliximab therapy was restarted due to clinical relapse in 21.1% (23/109) of patients after a median 4.8 month (interquartile range: 3.2–6.3 month). The median CDAI was 327 (interquartile range: 115–369) at the time of relapse. In UC, infliximab therapy needed to be restarted in 14.02% (15/107) patients after a median 6.7 month (interquartile range: 2.8–9.7 month). The median partial Mayo score was 5.9 (interquartile range: 4.5–7.2) at the time of retreatment. Of note, infliximab therapy was restarted in the 10.19% (22/216) patients (13 CD, 9 UC) who achieved mucosal healing, and 13.89% (30/216) patients (18 CD, 12 UC) who achieved clinical remission and 6.48% (14/216) patients (8 CD, 6 UC) who achieved full remission had to be retreated within the next year. Endoscopic activity was not assessed in each patient when the infliximab therapy was restarted. The response rates for retreatment were 78.26% (18/23) in CD and 66.67% (10/15) in UC within an average of three months after the reintroduction of infliximab therapy.

**Figure 1:**
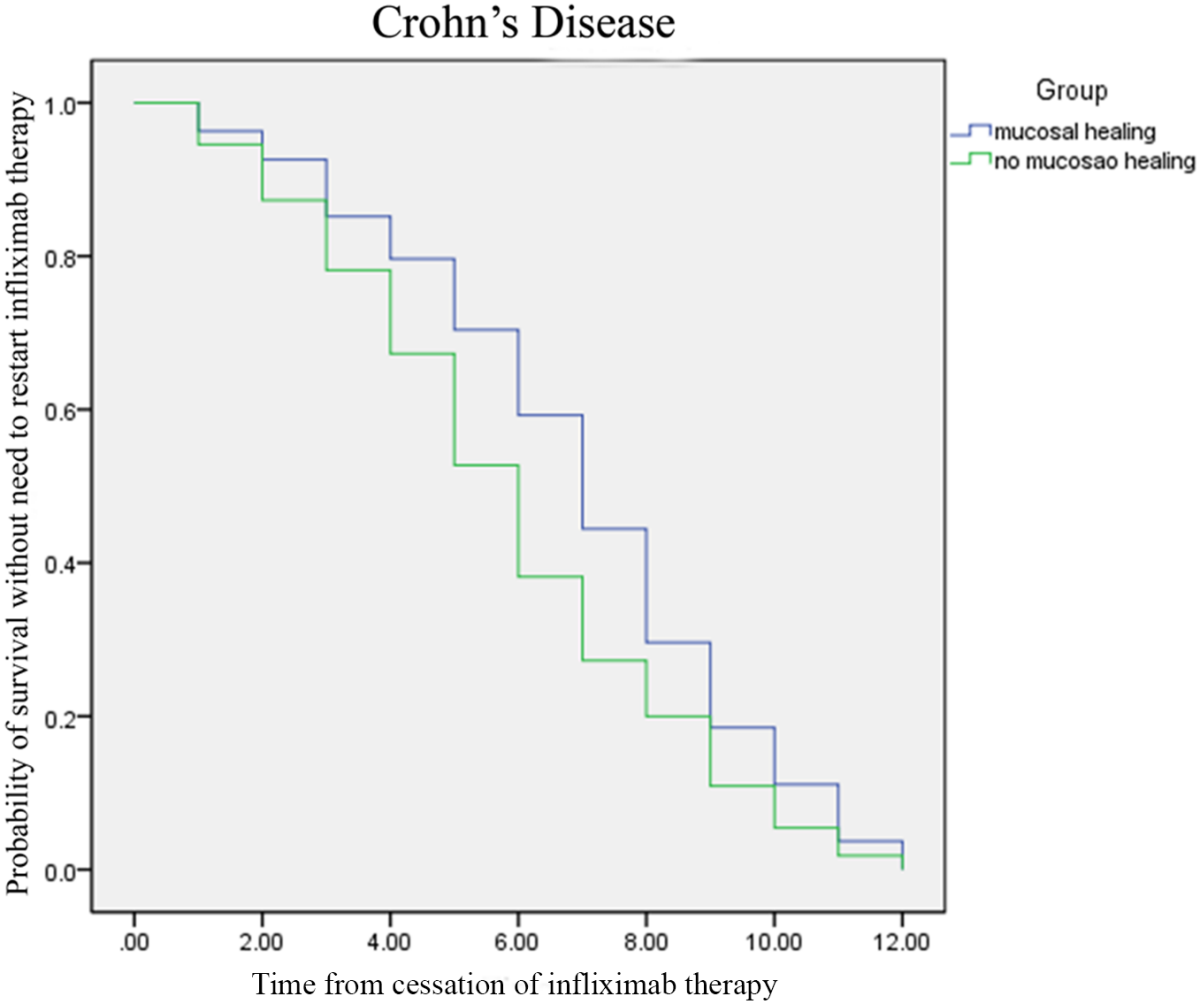
Kaplan-Meier analysis using Log-Rank and Breslow tests (clinical remission or mucosal healing was not associated with the time to restarting infliximab therapy in Crohn’s disease).

**Figure 2:**
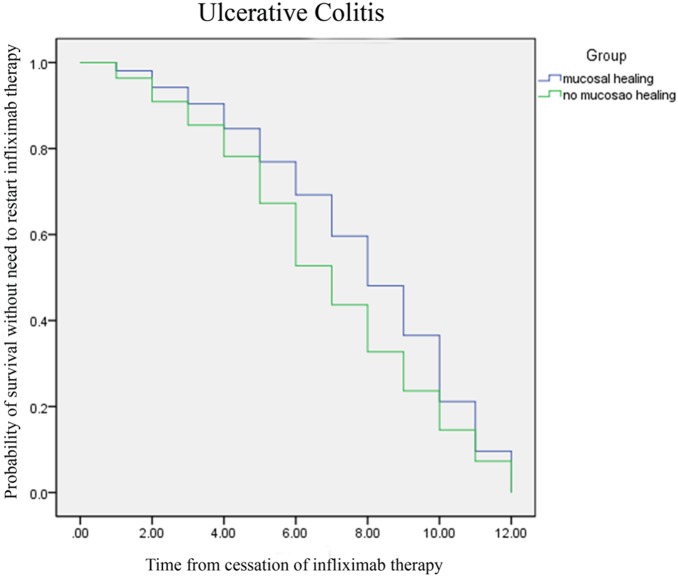
Kaplan-Meier analysis using Log-Rank and Breslow tests (clinical remission or mucosal healing was not associated with the time to restarting infliximab therapy in Ulcerative colitis).

In a univariate or Kaplan-Meier analysis using the Log-Rank and Breslow tests, neither clinical remission nor mucosal healing was associated with the time to restarting infliximab therapy in either CD (Figure 1) or UC (Figure 2). In univariate analysis or logistic regression analysis, none of the investigated parameters (e.g., gender, disease duration, smoking status, history of appendectomy, location/extent, behavior, extraintestinal manifestations, previous surgery, previous biological therapy, CRP level, or the effect of induction therapy) was associated with the need to restart infliximab therapy in either CD or UC (Table 2).

**Table 2:** Univariate regression analysis of need for retreatment with infliximab therapy.

Factor	*CD-P* value	*UC-P* value
Gender	0.87	0.63
Disease duration	0.12	0.15
Smoking status	0.96	0.53
Appendectomy	0.98	-
Location/extent	0.92	0.87
Behaviour	0.98	-
Extraintestinal manifestations	0.37	-
Steroid therapy at inclusion	0.19	0.13
Previous surgery	0.98	-
Previous biological therapy	0.42	-
Elevated CRP level	0.51	0.86
Outcome of induction therapy	0.29	0.23

## Discussion

In this prospective observational study conducted in patients with CD and UC receiving infliximab therapy for one year, mucosal healing was observed in 71.56% and 69.16% of the patients, respectively. Full remission, including both clinical and endoscopic remission, was detected in 56.88% and 54.21% of patients with CD and UC. Retreatment with infliximab therapy was necessary in 16.67% (13/78) of CD patients and in 12.16% (9/74) of UC patients, despite their achieving mucosal healing at the end of the year of infliximab therapy. Our results showed that mucosal healing after one year of infliximab treatment was not associated with sustained clinical remission.

Regarding the therapeutic approach of IBD, anti-TNF-α drugs proved to be the most effective in inducing mucosal healing. For example, The ACT trials confirmed the efficacy of infliximab in inducing and maintaining mucosal healing in active UC [20]. And The ACCENT I study confirmed that scheduled infliximab therapy is more effective in achieving mucosal healing than episodic treatment [21] for CD. In our study, infliximab therapy proved to be more effective in achieving mucosal healing in CD than in UC. This result may be due to the difference in the sizes of the inflamed area and the proportion of patients with previous biological therapy in the CD and UC groups. Now mucosal healing is very important for patients with IBD, because it seems to be associated with better outcomes (reduced rate of complications, surgery and hospitalization) in CD [21], [22]. In a Norwegian study, mucosal healing was associated with lower colectomy rate in UC and decreased need for steroid treatment in CD [23]. The STORI trial suggested that one of the most important predictors of relapse was the absence of mucosal healing at the time of drug withdrawal [14]. The study of Baert *et al* [24] confirmed that complete mucosal healing in patients with early stage CD predicted sustained steroid-free remission 3 and more years. At the same time, Ananthakrishnan have found that mucosal healing as an endpoint is cost effective in CD patients [25].

Now an important question is when mucosal healing should be established. International guidelines recommend assessing endoscopic mucosal healing before stopping the therapy with anti-TNF agents. But our results do not support the guidelines because more than 13.82% of the patients with mucosal healing relapsed and needed retreatment after one-year infliximab therapy. At the same time, there is no established guideline on when anti-TNF agents can be discontinued. Different studies have concluded different views. For example, In the STORI study, infliximab therapy was terminated in 115 CD patients in clinical remission after treatment with scheduled infliximab for at least one year [14]. But Forty-five percent of patients relapsed following withdrawal from infliximab. In a Danish single-center study, 24% of CD patients and 30% of UC patients discontinued infliximab while in clinical steroid-free remission [26]. The proportion of patients in remission declined steadily, with 61% of CD patients and 75% of UC patients remaining in remission after 1 year. Half of these patients maintained their remission after a median of 2 years. In total, 96% of CD patients and 71% of UC patients experienced complete clinical remission when retreated with infliximab after their relapses. In our study, the relapsed rate of the full remission patients was 11.67%, and the response rates for retreatment were 78.26% in CD and 66.67% in UC at the end of the year of infliximab therapy.

There are some limitations in our study. Firstly, biopsy samples were not taken routinely to assess microscopic activity of patients with IBD. Theoretically, the microscopic evaluation of the mucosa reflects the therapeutic response more accurately than an endoscopy, but it should be noted that the histological assessment of biopsy samples demonstrates only mucosal abnormalities. For example, the transmural pattern of CD is difficult to evaluate [27] by biopsy. And Laharie *et al* [28] did not find any correlation between histologically confirmed microscopic inflammation and endoscopic activity indices in patients with IBD. Therefore, the necessity for microscopic evaluation in the assessment of mucosal healing in IBD may be worthy of further consideration. Secondly, there is no widespread agreement regarding an acceptable definition of mucosal healing. The disappearance of mucosal ulcers and erosions may be used for mucosal healing more frequently in clinical practice. In our study, mucosal healing was evaluated on the basis of endoscopic activity indices. Finally, the sample size of our study is not particularly large, but we think that the tendency of our results is worth considering.

Now the primary goals of treatment in IBD are not only the induction and maintenance of clinical remission but also the induction of mucosal healing in an attempt to alter the course of IBD. And mucosal healing represents a more reliable and objective marker in the assessment of therapeutic response than clinical activity indices. Macroscopic findings of the mucosa represent its real alterations of patients with IBD, so endoscopy is the gold standard method of assessing mucosa lesions of patients with IBD.

But none of the studies support that mucosal healing correlates with clinical activity of IBD. Our results have revealed a high proportion of patients who relapsed even after mucosal healing. The higher relapse rates in patients with IBD who achieved mucosal healing may be explained by the shorter duration of their infliximab therapy. Therefore, the necessity of the routine endoscopic examinations at the end of the one-year period of infliximab therapy is worth considering. In conclusion, we think that stopping or continuing infliximab therapy may be determined by assessing the IBD patient’s general condition and the clinical activity. At the same time, we have concluded that the long-term advantages of mucosal healing can be achieved only if we continue previous effective therapies, even after mucosal healing by the endoscopic examination. In future, large controlled clinical trials are required to confirm these results.
